# The Effect of Warm-up on Tethered Front Crawl Swimming Forces

**DOI:** 10.2478/v10078-011-0066-1

**Published:** 2011-10-04

**Authors:** Henrique Neiva, Pedro Morouço, António J. Silva, Mário C. Marques, Daniel A. Marinho

**Affiliations:** 1 Department of Sport Sciences, University of Beira Interior (UBI), Covilhã, Portugal; 2 Department of Sport Sciences, Exercise and Health, University of Trás-os-Montes and Alto Douro (UTAD), Vila Real, Portugal; 3 Polytechnic Institute of Leiria. Research Center for Human Movement Sciences (IPL), Leiria, Portugal; 4 Research Centre in Sports Sciences, Health and Human Development (CIDESD), Vila Real, Portugal

**Keywords:** evaluation, strength, performance, lactate, perceived exertion

## Abstract

This study was conducted to determine the effect of warm-up on high-intensity front crawl tethered swimming and thus to better understand possible variations in the force exerted by the swimmers. Ten male national level swimmers (mean ± SD; age 15.3 ± 0.95 years old, height: 1.73 ± 5.2 m, body mass: 64.3 ± 7.8 kg, Fat mass 8.31 ± 3.1 kg) participated in this study. After a typical competition warm-up, the subjects performed a 30 s tethered swimming all-out effort in front crawl swimming technique. The same test was repeated in the day after but performed without warming up. Capillary blood lactate concentration was assessed before and after the swimming test and the Borg ratings of perceived exertion scale was used. Without a previous warm-up, the mean ± SD values of maximum and mean forces were 299.62 ± 77.56 N and 91.65 ± 14.70 N, respectively. These values were different (p<0.05) from the values obtained with warm-up (351.33 ± 81.85 N and 103.97 ± 19.11 N). Differences were also observed when regarding to the forces relative to body mass. However, the values of lactate net concentrations after the test performed with and without warm-up were not different (6.27 ± 2.36 mmol·l^−1^ and 6.18 ± 2.353 mmol·l ^−1^) and the same occurs with the values of ratings of perceived exertion (15.90 ± 2.42 and 15.60 ± 2.27). These results suggest an improvement of the maximum and mean force of the swimmer on the tethered swimming due to previous warm-up.

## Introduction

Warm-up procedures before competition or training are intended to assure benefits to athlete’s performance (Atkinson et al., 2005; Burnley et al., 2002) Although there are few data available on physiological responses to the warm-up, these routines are well accepted and commonly used by athletes and their coaches (Bishop, 2003). For example, the mechanisms related to the raise of core and muscle temperature seem to be of great importance for the proposed effects of warming-up before physical activity (Asmussen and Boje, 1945). Temperature might improve performance by decreasing the viscous resistance of muscles and joints (Wright and Johns, 1961; Cavagna, 1993), increasing of nerve conduction rate and speeding of metabolic reactions, such as the muscle glycogenolysis, glycolysis and high energy phosphate degradation (Febbraio et al., 1996). This temperature rise, due to the warming-up routines performed, might also contribute to increase the oxygen delivery to the muscles, via a rightward shift in the oxyhaemoglobin dissociation curve and vasodilatation of muscle blood vessels (McCutheon et al., 1999). Beyond this temperature-related mechanism, warm-up seems to allow the athletes to begin subsequent tasks with an elevated baseline of VO_2_, leaving more anaerobic capacity for later in the task (Febraio et al., 1996). Post activation potentiation (Sale, 2002) is also presented to be responsible for a better performance after warming-up procedures.

Despite there were several studies demonstrating improvements in performance after warming-up (Andzel, 1982; Asmussen and Boje, 1945; Atkinson et al., 2005; Burnley et al., 2002), there were others reporting no changes or even detrimental changes in performance (Andzel, 1982; Bruyn-Prevost and Lefebvre, 1980; Mitchell and Huston, 1993; Bishop et al., 2001). Thus, there is still some inconsistency in this matter, and more studies are needed to further determine the importance of warm-up procedures, their effect in performance or even their optimal structure, especially in each sport specificity (Fradkin et al., 2010). Possibly, because of the particular environment, swimming warm-up related studies are very scarce.

The main aim of the swimmers is to perform a prescribed distance in the shortest time possible, according to the rules established. In this way, the force produced by the swimmer, needed to overcome drag and to increase the swimming velocity, seems to be extremely relevant (Smith et al., 2002; Marinho et al., 2010). This force can be evaluated by dry-land strength and power tests (Garrido et al., 2010). However, the tethered swimming is proposed to specifically assess its interaction with swimming technique (Keskinen, 1994). Full or partial tethered swimming has been recognized as a useful tool to measure the force exerted by a swimmer (Magel, 1970; Yeater et al., 1981; Costill et al., 1986; Filho and Denadai, 2008). This method was firstly introduced by Magel (1970), who evaluated the four swimming techniques and suggested breaststroke to have the highest values of force production. Used as an adaptation of the Wingate test (Stager and Coyle, 2005), the tethered swimming can be performed in water as a more specific ergometer. The swimmer is connected to the wall by an elastically (partial tethered) or non-elastic cable (full tethered) and produces a maximal effort, using an apparatus that measures the force produced as a biokinetic bench (Costill et al., 1983) or a strain gauges system (Morouço et al., 2011). This is a specific test for swimmer′s anaerobic evaluation and has been pointed as a measurement of maximum propulsive force that corresponds to the resultant force needed to overcome the resistance at maximum swimming velocity (Clarys, 1979; Keskinen, 1994).

Therefore, the aim of the current study was to compare the force exerted by the swimmer during tethered swimming with and without warming-up and to understand the effects of warm-up in the propulsive force produced by the swimmer.

## Material and Methods

### Subjects

Ten male swimmers (mean ± SD; age 15.3 ± 0.95 years-old, height: 1.73 ± 5.2 m, body mass: 64.3 ± 7.8 kg, fat mass 8.31 ± 3.1 kg) participated in this study. Body mass and fat mass were assessed through a bioelectric impedance analysis method (Tanita BC 420S MA, Japan). Their training experience was of 7.2 ± 1.1 years, training from 6 to 9 times a week and all of them are national level swimmers, participating in National Championships. The participants’ parents and coaches provided written informed consent to participate in this research, and the procedures were approved by the institutional review board.

### Testing procedures

The experiments were performed in a 50 m indoor swimming pool at a water temperature of 27.5ºC. The data collection was implemented one week after the main competition (National Championships) of the season second macrocycle. Swimmers were involved in two similar protocols of tethered front crawl swimming, one executed with a previous warm-up, and another without warm-up procedures. The warm-up procedures (dry and in-water) consisted of their typical warm-up frequently performed before a competitive swimming event (total volume: 1000 m). After 10 min rest, the tethered swimming protocol was implemented. One day after, the same protocol was repeated, but without warming up. The swimmers were wearing a belt attached to a steel cable (negligible elasticity). As the force vector in the tethered system presented a small angle to the horizontal, computing the horizontal component of force, data was corrected. A load-cell system connected to the cable was used as a measuring device, recording at 100 Hz with a measure capacity of 5000 N. The data obtained was transferred by a Globus Ergometer data acquisition system (Globus, Italy) that exported the data in ASCII format to a computer. Individual force to time F (t) curves were assessed and registered to obtain maximum force (Fmax, the highest value of force produced in first 10 s) absolute and relative values and; mean force (Fmean – average force values during the 30s test) absolute and relative values. The test started after an acoustic signal, with the swimmers in a horizontal position, with the cable fully extended. The data collection started after the first stroke cycle to avoid the inertial effect of the cable extension after the first propulsion.

The swimmers swam as natural as possible during 30 s, at maximum intensity. Additionally, capillary blood samples were collected from the fingertip before and after each tethered swimming (at the 1^st^ and 3^rd^ min of recovery) to access the higher values of blood lactate concentration ([La-]) (Accutrend Lactate^®^Roche, Germany). The values of [La-]net were determined by the difference between [La-] after the test and the resting values. The Borg (1998) ratings of perceived exertion (RPE) scale was used to quantify exercise level of exertion after each test.

### Statistics

Standard statistical methods were used for calculation of means and standard deviations. Normality was determined by Shapiro-Wilk test. Since, the very low value of the N (i.e., N < 30) and the rejection of the null hypothesis (H_0_) in the normality assessment, non-parametric procedures were adopted. In order to compare the data obtained with and without warm-up, non-parametric Wilcoxon signed rank test was used. Differences were considered significant for p ≤ 0.05.

## Results

Table 1 presents the mean ± SD values for the tethered absolute variables, namely the maximum force and mean force. Significant differences were evident for the data obtained on tethered front crawl swimming test after warm-up and without warm-up. The warm-up condition presented higher values.

**Figure 1:**
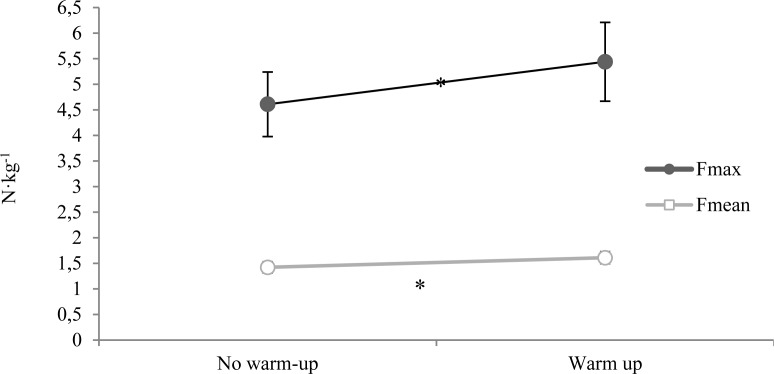
Mean ± SD values of maximum (Fmax) and mean forces (Fmean) relative to the weight of the swimmers, exerted during tethered swimming test. * Represents significant differences (p ≤ 0.01) between tests performed without warm-up and with warm-up.

Figure 1 presents relative values of the maximum and mean forces in both conditions. The body mass of the swimmers were used to determinate these relative forces, and the graphic demonstrates the differences between the values obtained (4.61 ± 0.63 N·kg^−1^ and 5.44 ± 0.77 N·kg^−1^, for Fmax without and with warm-up; 1.42 ± 0.12 N·kg^−1^ and 1.61± 0.13 N·kg^−1^ for Fmean without and with warm-up, respectively).

Additionally, table 2 presents the mean ± SD values of the ratings of perceived exertion scale and the values of blood lactate concentration attained after the swimming test in both conditions.

## Discussion

The aim of this research was to investigate the effect of the warm-up in the force exerted on the tethered front crawl swimming in high-level swimmers. Main results suggest an improvement of the maximum and mean force of the swimmer on the tethered swimming due to previous warm-up.

In a broad sense, warm-up is used to increase muscle and tendon mobility, to stimulate blood flow, to increase muscle temperature and to improve coordination (Smith, 2004). Although the great importance placed in warm-up procedures by coaches and their athletes, it is a fact that their effects or even their ideal structure or type, are not well-known. Specifically in swimming, the literature is very scarce on this matter and uses different methodologies, which makes difficult the comparison between results and emphasizes the need for more researching (Fradkin et al., 2010).

The tethered swimming is a methodology that allows obtaining data information related with propulsive force that swimmers can exert in their specific environment. The procedures used provide a continued measurement and recording of propelling force exerted during swimming (Mouroço et al., 2011). The Fmax absolute values obtained for front crawl were higher than those presented by the specialized literature. These differences could be due to different methodology used (Keskinen, 1997) or even because our sample contained subjects from only one gender (Morouço et al., 2011). Higher values of Fmax relative, Fmean absolute and relative were also observed when comparing to the results obtained by Morouço et al. (2011). Considering the data presented by the previous authors, Fmean absolute value without warm-up was the only value of force of the current study that is similar to the literature (92.8 ± 33.7 N). Moreover, it is important to notice that the values of force obtained (absolute and relative) were higher when the swimmers performed a previous warm-up as they usually do before swimming events. When warming-up before the tethered front crawl swimming, swimmers exerted 14.72 ± 0.13% additional maximum force and 11.52 ± 0.05% additional mean force than with no warming-up (Fig. 1). These results reveal the positive effect of warm-up procedures on the propulsive forces (maximum and mean values) produced by the swimmers, suggesting the high importance of these warm-up routines.

Regarding to the ratings of perceived exertion scale, there were no differences between the two conditions of the test in the present research. This indicator is an important complement to physiological measurements, presenting strong relationships with some of these parameters. It is a measure used to quantify, monitor and assess an individual’s exercise level of exertion (Borg, 1998). Despite there were no significant differences between the effort made with and without a previous warm-up, the average value of RPE obtained without warm-up appeared to be slightly higher. This suggests a tendency of a superior perceived effort by the swimmers when performing the tethered test in this condition. However, more research is needed to clarify this parameter.

The warm-up is proposed to maintain the acid-base balance at an appropriate level by stimulating the buffering capacity (Beedle et al., 2007; Mandegue et al., 2005). Poprzecki et al. (2007) presented differences in [La-] values between the Wingate test performed with and without previous warm-up. Despite this result, in the present study the values of [La-]net obtained after the tethered swimming revealed no differences between the two conditions (no warm-up vs. warm-up). [La-] values had been commonly used to estimate the anaerobic capacity of the athlete and the contribution of the glycolytic metabolism to exercise (di Prampero et al., 1999). Considering that the values of resting [La-] were removed to the data presented, [La-]net values obtained confirmed the high anaerobic contribution to perform this 30 s tethered front crawl swimming test.

To the best of our knowledge, this study was the first to compare the forces exerted by the swimmers in their specific environment with and without a previous warm-up. The measurements of force production exerted in the water are a reliable method to evaluate the capacity of the swimmer to use muscular strength in effectively propulsive force (Costill et al., 1986). Moreover, although tethered swimming is different from free swimming, it seems to be a better methodology to estimate propelling forces than dry-land testing protocols, based on the significant correlation between average maximum force and swimming velocity (Keskinen, 1997).

In conclusion, the present study revealed that the warm-up seems to improve the maximum and mean propelling forces of the swimmer in front crawl swimming technique, registering no differences in the [La-]net values and in the ratings of perceived exertion. The high relationships between the 30 s tethered swimming test and swimming performance (Morouço et al., 2011) lead us to hypothesize a positive effect of the warm-up in performance. Nevertheless, further research is needed to continue exploring this important scope in sports performance that remains controversial and relatively unknown.

## Figures and Tables

**Table 1:** Mean ± SD values of maximum (Fmax) and mean forces (Fmean) exerted during the tethered swimming test. P-values are presented

	**No warm-up**	**Warm-up**	**p values**
**Fmax (N)**	299.62 ± 77.56	351.33 ± 81.85	p = 0.009
**Fmean (N)**	91.65 ± 14.70	103.97 ± 19.11	p = 0.005

**Table 2:** Ratings of perceived exertion scale (RPE) (mean ± SD) and difference between pre and post blood lactate concentration values ([La-]net) ( mean ±SD). P-values are also presented

	**No warm-up**	**Warm-up**	**p values**
**RPE**	15.90 ± 2.42	15.60 ± 2.27	p = 0.496
**[La-]net (mmol·l^−1^)**	6.27 ± 2.36	6.18 ± 2.35	p = 0.767
